# Homozygous *GDF2* nonsense mutations result in a loss of circulating BMP9 and BMP10 and are associated with either PAH or an “HHT‐like” syndrome in children

**DOI:** 10.1002/mgg3.1685

**Published:** 2021-04-09

**Authors:** Joshua Hodgson, Lidia Ruiz‐Llorente, Jamie McDonald, Oliver Quarrell, Kelechi Ugonna, James Bentham, Rebecca Mason, Jennifer Martin, David Moore, Katie Bergstrom, Pinar Bayrak‐Toydemir, Whitney Wooderchak‐Donahue, Nicholas W. Morrell, Robin Condliffe, Carmelo Bernabeu, Paul D. Upton

**Affiliations:** ^1^ Department of Medicine University of Cambridge Cambridge UK; ^2^ Centro de Investigaciones Biológicas Margarita Salas Consejo Superior de Investigaciones Científicas (CSIC), and Centro de Investigación Biomédica en Red de Enfermedades Raras (CIBERER) Madrid Spain; ^3^ Department of Systems Biology School of Medicine and Health Sciences University of Alcalá Madrid Spain; ^4^ HHT Center Department of Pathology University of Utah Salt Lake City UT USA; ^5^ Department of Clinical Genetics Sheffield Children’s Hospital Sheffield UK; ^6^ Department of Respiratory Medicine Sheffield Children’s Hospital Sheffield UK; ^7^ Department of Paediatric Congenital Heart Disease Leeds Children’s Hospital Leeds UK; ^8^ NHS Lothian Molecular Genetics Service Western General Hospital Edinburgh UK; ^9^ Department of Pediatrics Baylor College of Medicine and Texas Children’s Hospital Houston TX USA; ^10^ ARUP Laboratories University of Utah School of Medicine Salt Lake City UT USA; ^11^ Sheffield Pulmonary Vascular Disease Unit Royal Hallamshire Hospital Sheffield UK

**Keywords:** bone morphogenetic protein, hereditary hemorrhagic telangiectasia, pulmonary arterial hypertension, pulmonary arteriovenous malformations

## Abstract

**Background:**

Disrupted endothelial BMP9/10 signaling may contribute to the pathophysiology of both hereditary hemorrhagic telangiectasia (HHT) and pulmonary arterial hypertension (PAH), yet loss of circulating BMP9 has not been confirmed in individuals with ultra‐rare homozygous *GDF2* (BMP9 gene) nonsense mutations. We studied two pediatric patients homozygous for *GDF2* (BMP9 gene) nonsense mutations: one with PAH (c.[76C>T];[76C>T] or p.[Gln26Ter];[Gln26Ter] and a new individual with pulmonary arteriovenous malformations (PAVMs; c.[835G>T];[835G>T] or p.[Glu279Ter];[Glu279Ter]); both with facial telangiectases.

**Methods:**

Plasma samples were assayed for BMP9 and BMP10 by ELISA. In parallel, serum BMP activity was assayed using an endothelial BRE‐luciferase reporter cell line (HMEC1‐BRE). Proteins were expressed for assessment of secretion and processing.

**Results:**

Plasma levels of both BMP9 and BMP10 were undetectable in the two homozygous index cases and this corresponded to low serum‐derived endothelial BMP activity in the patients. Measured BMP9 and BMP10 levels were reduced in the asymptomatic heterozygous p.[Glu279Ter] parents, but serum activity was normal. Although expression studies suggested alternate translation can be initiated at Met57 in the p.[Gln26Ter] mutant, this does not result in secretion of functional BMP9.

**Conclusion:**

Collectively, these data show that homozygous *GDF2* mutations, leading to a loss of circulating BMP9 and BMP10, can cause either pediatric PAH and/or “HHT‐like” telangiectases and PAVMs. Although patients reported to date have manifestations that overlap with those of HHT, none meet the Curaçao criteria for HHT and seem distinct from HHT in terms of the location and appearance of telangiectases, and a tendency for tiny, diffuse PAVMs.

## Graphical abstract

Ultra‐rare homozygous GDF2 mutations have not previously been shown to lead to loss of circulating BMP9 protein. We show that homozygous GDF2 mutations in two pediatric individuals from two different families, lead to loss of plasma BMP9 and BMP10, resulting in a loss of serum‐derived BMP‐dependent endothelial signaling. The two individuals have different vascular dysplasias: one with pulmonary arterial hypertension and the second with a pulmonary arteriovenous and telangiectasia syndrome that is distinct from hereditary hemorrhagic telangiectasia but may be mistakenly diagnosed as hereditary hemorrhagic telangiectasia.

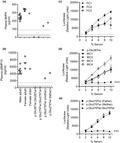

## 1 INTRODUCTION

Hereditary hemorrhagic telangiectasia (HHT1: [OMIM 183700] and HHT2: [OMIM 600376]) and pulmonary arterial hypertension (PAH [OMIM 178600]) are vascular dysplasias characterized by disparate pathologies. Recognizing that the vascular malformations of HHT did not occur randomly, the consensus clinical diagnostic (Curaçao) criteria were agreed (Shovlin et al., 2000), namely: (a) spontaneous and recurrent epistaxis; (b) multiple telangiectases in characteristic locations (lips, oral cavity, fingers, and nose); (c) visceral (gastrointestinal, pulmonary, hepatic, brain, and spinal) arteriovenous malformations (AVMs); and (d) first‐degree relative with HHT according to these criteria. The diagnosis is considered definite if three or more criteria are present, possible if two criteria are present, and unlikely if one is present (Shovlin et al., 2000). However, as the typical age of onset for recurrent epistaxis and multiple oral/cutaneous telangiectases is late childhood, HHT is not ruled out by the lack of three or more diagnostic criteria in children (Govani & Shovlin, 2009). Pathologically HHT is characterized by telangiectases and arteriovenous malformations (AVMs), the latter representing dilated tortuosities forming direct links between arteries and veins, thus bypassing the capillary bed. For example, pulmonary AVMs (PAVMs) can cause anatomical right‐to‐left shunting of the blood, resulting in hypoxemia and several other complications (Shovlin, 2010, 2014). Contrasting with the widespread organ pathologies of HHT, PAH primarily affects the lung vasculature. The pathology of PAH is characterized by narrowing and occlusion of pulmonary arterioles due to excessive proliferation and hypertrophy of cells in the vessel intima, media, and adventitia (Archer et al., 2010). The combination of increased muscularization and vasoconstriction underlies the elevated resting mean pulmonary artery pressure (>25 mmHg) and pulmonary vascular resistance that overloads the right ventricle (Thenappan et al., 2018). Patients experience worsening dyspnea and eventually die from right‐heart failure (Thenappan et al., 2018). Although HHT and PAH differ pathologically, a subset of HHT patients exhibit either post‐capillary high‐output pulmonary hypertension or pre‐capillary PAH as comorbidities, though the relative prevalence of these has not been comprehensively assessed (Vorselaars et al., 2015).

Intriguingly, the genes identified with deleterious mutations in HHT and PAH patients overlap considerably and collectively that suggests disruption of endothelial BMP9/10 signaling. BMP9 and BMP10 are circulating BMPs that bind and activate high affinity endothelial receptor complexes comprising ALK1 and BMPR‐II or ACTR‐IIA, utilizing endoglin as a co‐receptor (David et al., ,^2007^, ^2008^; Scharpfenecker et al., 2007; Upton et al., 2009). The activated ALK1–BMPR‐II complex mediates signaling via the canonical Smad1/5 proteins in complex with Smad4. Smad9 may contribute to canonical signaling and/or function as an inhibitory Smad (Tsukamoto et al., 2014). Ultimately, this signaling axis is considered to maintain normal endothelial homeostasis by promoting endothelial quiescence and reducing apoptosis and barrier permeability (David et al., 2008; Long et al., 2015). The contribution to HHT and PAH of heterozygous mutations in members of this pathway is well founded. Mutations in endoglin (*ENG* [OMIM 131195] and activin A receptor‐like type 1 (*ACVRL1* or ALK1 [OMIM 601284]) cause HHT type 1 and HHT type 2, respectively. Mutations in these genes account for up to 96% of all HHT cases (McDonald et al., 2020). Furthermore, Smad4 (*SMAD4* [OMIM 600993]) mutations cause a syndrome of combined Juvenile polyposis and HHT (JP‐HHT; McDonald et al., 2020). In PAH, mutations in *BMPR2* [OMIM 600799], encoding the bone morphogenetic type‐II receptor (BMPR‐II), account for 53%–86% of familial PAH cases and 14%–35% of idiopathic cases (Machado et al., 2015). In a small proportion of PAH patients, mutations have been identified in *ACVRL1*, *ENG*, *SMAD9* [OMIM 603295] and the genes encoding BMP9 (*GDF2* [OMIM 605120]) and BMP10 (*BMP10* [OMIM 608748]; Drake et al., 2011; Eyries et al., 2019; Gräf et al., 2018; Machado et al., 2015; Shintani et al., 2009; Wang et al., 2019). We also previously reported the association of heterozygous missense BMP9 mutations with a vascular anomaly syndrome characterized by cutaneous telangiectases and AVMs (Wooderchak‐Donahue et al., 2013).

BMP9 is primarily expressed in the liver (Bidart et al., 2012; Miller et al., 2000) whereas BMP10 is highly expressed not only in the right atrium, but is also expressed at low levels in the liver (Chen et al., 2004; Jiang et al., 2016; Tillet et al., 2018). BMP9 and BMP10 are secreted as dimers of their growth‐factor domains (GFDs) in a non‐covalent complex with their respective prodomains (Pro:BMP9 and Pro:BMP10; Bidart et al., 2012; Jiang et al., 2016). However, the majority of plasma BMP10 may be inactive protein secreted without cleavage of the polypeptide chain between the prodomain and growth factor domain (*ProBMP10*; Hodgson et al., 2019). Recent evidence has also suggested the presence of circulating BMP9/BMP10 heterodimers, possibly produced by the liver (Tillet et al., 2018). Heterozygous germ‐line BMP9 (*GDF2*) missense, truncating, and deletion mutations have been reported in patients with PAH on four separate occasions, bringing the total number of reported cases to 32 (Eyries et al., 2019; Gräf et al., 2018; Wang et al., 2016; Wang et al., 2019). *In vitro* analysis confirmed that the secretion and/or function of these PAH‐related BMP9 mutant proteins was impaired, and BMP9 levels and activity were reduced in patient plasmas (Gräf et al., 2018; Hodgson et al., 2019; Wang et al., 2019). A pediatric PAH patient harboring a homozygous nonsense p.[Gln26Ter] BMP9 mutation, predicted to lead to an absence of BMP9 protein, has been reported but BMP9 levels were not assessed in this patient (Wang et al., 2016). During the preparation of this manuscript, a family with a pediatric HHT case with PAVMs harboring a p.[Tyr354ArgfsTer15];[Tyr354ArgfsTer15] *GDF2* mutation was reported, although measurements of BMP9/10 levels and activity were not undertaken (Liu et al., 2020). A recent large‐scale RNA‐seq study has suggested that up to 68% of variants predicted to be degraded by nonsense‐mediated decay avoid RNA surveillance and lead to full length transcripts (Lappalainen et al., 2013). Therefore, the presence of a premature truncation codon in *GDF2* is not a proof of protein loss.

Here we characterize the plasma levels of BMP9 and pBMP10 and serum‐derived endothelial BMP activity in two pediatric patients homozygous for *GDF2* nonsense mutations, a PAH patient (p.[Gln26Ter];[Gln26Ter]) and a second patient (p.[Glu279Ter];[Glu279Ter]) who exhibit a phenotype of PAVMs and cutaneous telangiectases, with no evidence of PAH. Although there is some overlap of this disease phenotype with HHT, careful examination of the phenotype suggests this is a distinct “HHT‐like” syndrome.

## 2 MATERIALS AND METHODS

### 2.1 Ethical compliance

This study was approved by the following ethical review boards: IRB_00020480 (Utah) and NIHR BioResource for Rare Diseases PAH Project (Sheffield). Informed consent was obtained from all human subjects in this study, though both families specifically refused consent for the inclusion of photographs of external features.

### 2.2 Blood samples

After informed consent was given, blood samples were collected into EDTA tubes for plasma preparation and serum separator tubes for serum. Samples were centrifuged within 30 min of collection. The plasma and serum fractions were then removed from their respective tubes and stored at −80°C until assay.

### 2.3 Plasmid generation and amplification

The plasmid pcDNA3.1/V5‐His‐TOPO/human BMP9‐WT (Wooderchak‐Donahue et al., 2013) was used as a template for site‐directed mutagenesis (GenScript, Leiden, Netherlands) to generate the c.[76C>T] mutation leading to the pcDNA3.1/V5‐His‐TOPO/human BMP9‐Q26X (ProBMP9‐p.[Gln26Ter]) construct. The human BMP9 sequence from the methionine residue at position 57 (Met57) to the stop codon at position 429 was amplified using pcDNA3.1/V5‐His‐TOPO/human BMP9‐WT as the template, and the resulting DNA fragment was subsequently cloned into pcDNA3.1/V5‐His‐TOPO to obtain pcDNA3.1/V5‐His‐TOPO BMP9(57‐429; designated Orf57), using the Gibson recombination cloning method (GenScript). Plasmids were then transformed into One Shot^®^ TOP10 Chemically Competent *E*. *coli* cells and clones were selected and amplified. Plasmids were purified using PureLink™ HiPure Plasmid Filter Maxiprep Kit (ThermoFisher). The resulting constructs were confirmed by Sanger sequencing using T7 and BGH primers.

### 2.4 Cell culture

Human embryonic kidney 293 T cells were cultured in Dulbecco's modified Eagle's medium (ThermoFisher) supplemented with 10% fetal bovine serum (FBS; ThermoFisher) and antibiotic/antimycotic (Invitrogen 15240‐062; ThermoFisher). HMEC‐1 human microvascular endothelial cells stably expressing the BRE‐luciferase reporter (HMEC‐1‐BRE) generated and cultured as described previously (Hodgson et al., 2019).

### 2.5 BMP9 ELISA

The BMP9 ELISA detects the free BMP9 GFD and Pro:BMP9, but not *ProBMP9* (Hodgson et al., 2019). For plasma BMP9 measurements, high binding 96‐well ELISA plates (Greiner) were coated with 0.5 μg/well anti‐human BMP9 (MAB3209, R&D Systems) in PBS (ThermoFisher), and incubated overnight at 4°C in a humidified chamber. Plates were washed with PBS containing 0.05% Tween 20 (PBS‐T), followed by blocking with 1% (w/v) bovine serum albumin (BSA; ThermoFisher) in PBS (PBS/1% BSA) for 2 hr at RT. Plasma samples (25 or 50 μL) were premixed with 50 μL PBS/1% BSA or BioRad diluent buffer (BUF037; BioRad), both then supplemented to give final concentrations of 0.5% Triton X‐100 and 0.2% goat serum (GS) in a total volume of 100 μL. Recombinant human BMP9 standards (4.88–5000 pg/ml) were prepared in the same final concentrations of additives. After washing, biotinylated anti‐human BMP9 detection antibody (0.04 µg/well BAF3209; R&D Systems) was added in PBS/1% BSA containing 0.2% GS. After further washing, ExtrAvidin(r)‐alkaline phosphatase (E2636; Sigma Aldrich) diluted 1:400 in PBS/1% BSA was added. The ELISA was developed with 1 mg/ml 4‐nitrophenyl phosphate disodium salt hexahydrate (N2640; Sigma Aldrich) in 1 M diethanolamine/0.5 mM MgCl_2_, pH 9.8, and absorbance measured at 405 nm. Unknown values were extrapolated from the standard curve using a four‐parameter log curve fit. All values are presented as the concentration of the BMP9‐GFD. The protocol using 25 μL plasma and BioRad diluent was found to be most sensitive, with a detection limit of 26.46 pg/ml and lower limit of quantification of 88.2 pg/ml.

For measurement of BMP9 in serial dilutions of conditioned media, 0.2 μg/well of MAB3209 was used and the Triton X‐100 and goat serum supplements were omitted. All samples were measured in duplicate.

### 2.6 pBMP10 ELISA

The pBMP10 ELISA detects Pro:BMP10 and *ProBMP10*, but not the BMP10 GFD (Hodgson et al., 2019). For plasma pBMP10 measurements, plates were coated with 0.5 µg/well of anti‐human BMP10 (MAB2926; R&D Systems) and blocked as above. Plasma samples (30 μL) were premixed with 70μL BioRad diluent, supplemented to give final concentrations of 0.5% Triton X‐100, 0.2% GS, and 4.5 mM EDTA. Dilutions of the furin‐cleaved purified Pro:BMP10 standard (97.65–100000 pg/ml GFD equivalent), produced as described previously (Jiang et al., 2016), were prepared to give the same final concentrations of additives. After washing, biotinylated anti‐human BMP10 propeptide (0.04 µg/well BAF3956; R&D Systems) in 1% BSA, 0.2% goat serum was added. Assays were then processed as described for BMP9. All data are presented as the concentration of the GFD component. All samples were measured in duplicate. The limit of detection of was 445.2 pg/ml and lower limit of quantification was 1484 pg/ml.

### 2.7 BMP activity assays

HMEC‐1‐BRE cells were grown to confluence in 96‐well plates. They were then quiesced in serum‐free MDCB 131 media overnight. Cells were then stimulated with patient serum (2%, 4%, 6%, 8%, and 10% by volume in serum‐free media) for 6 hr. Cells were lysed with 100 µL OneGlo reagent (Promega), samples were transferred to a white plate, and luciferase activity was measured by a luminescence reader. Samples were measured in duplicate and assays using serum were found to be most sensitive and reliable as plasma was prone to clotting at 2%–3% by volume.

### 2.8 BMP9 overexpression

HEK‐EBNA cells (ATCC; Manassas, VA) were grown to confluence in 6 cm dishes, and then washed with Optimem. Cells were transfected with plasmids (2 µg) and Lipofectamine 2000 (15 µL) according the manufacturer's instructions. After 5 hr, the transfection mix was replaced with 3 ml of serum‐free chemically defined Chinese hamster cell over‐expression media (CDCHO, ThermoFisher). After 3 days, conditioned media were collected, cleared by centrifugation, aliquoted, and frozen at −80°C. Cell lysates were collected in SDS lysis buffer (125 mM Tris, pH7.4, 10% glycerol, and 2% SDS) supplemented with an EDTA‐free protease inhibitor (Roche). Three independent batches of cells were transfected and harvested. The activity of the Pro:BMP9‐WT control was identical to previously validated Pro:BMP9‐WT (Wei et al., 2014) with respect to endothelial cell activity, cross‐reactivity by ELISA, and Western blotting.

### 2.9 Western Blotting

Cell lysates were thawed, sonicated at 10 µm amplitude for 10 s, and then cleared by centrifugation. A modified Lowry assay was carried out (BioRad) to quantify the protein concentration of each sample. Conditioned media (60 µL) or lysates (30 µg of total protein) were mixed with 5x SDS loading buffer (312.5 mM Tris–HCl, pH 6.8, 10% SDS, 50% glycerol, 0.0075% bromophenol blue, and 12.5% β‐mercaptoethanol) and boiled at 98°C for 5 min to denature and reduce proteins. Samples were then analyzed by gel electrophoresis and western blotting. For the growth factor domain, samples were blocked in 5% BSA and probed using sc‐514211 (1:1000; Santa Cruz Biotechnology). For the prodomain, samples were blocked in 5% non‐fat milk protein (Marvel) and probed using AF3879 (1:2500; R&D Systems). Lysate blots were re‐probed using an antibody toward β‐actin (A5441, clone AC‐15; Sigma Aldrich).

## 3 RESULTS

### 3.1 Phenotypic characteristics of individuals with homozygous *GDF2* mutations

Two individuals from different families were identified as harboring homozygous germ‐line nonsense mutations in the *GDF2* gene as described below:

Individual 1: (NM_016204.3:c.[76C>T];[76C>T] or p.[Gln26Ter];[Gln26Ter]) has pulmonary arterial hypertension (PAH) and multiple spider‐like telangiectases were noted on his face bilaterally during his most recent evaluation at 10‐years of age. A targeted full body exam for cutaneous vascular lesions was otherwise negative. He has no history of even minor recurring epistaxis or other bleeding. An agitated saline contrast echocardiogram was negative for evidence of a pulmonary shunt/PAVM.

He initially presented at age 3 to Wang et al., (2016) with a severely dilated right ventricle and an estimated systolic pulmonary arterial pressure of >100 mmHg by echocardiography. He responded to PAH therapy in the form of sildenafil (20 mg, 3 times a day), bosentan (62.5 mg, twice daily), and subcutaneous treprostinil. Right heart catheterization after 3 months of therapy demonstrated mild‐to‐moderate PAH with a mean pulmonary arterial pressure of 30 mmHg, a normal pulmonary arterial wedge pressure of 7 mmHg, cardiac output via the indirect Fick method of 5.2 L/min/m^2^, and pulmonary vascular resistance of 4.4 Wood Units. No clinical features of HHT were noted at this time. Given the diagnosis of PAH, the *KCNK3* (OMIM 603220), *KCNA5* (OMIM 176267), *CAV1* (OMIM: 601047), *BMPR2*, *SMAD4*, *ENG*, and *ACVRL1* genes were also sequenced, with no pathogenic or suspicious variant detected. The homozygous *GDF2* variant was reported as the suspected cause of his PAH (Wang et al., 2016), but without detailed evaluation of the patient or parents for features of HHT or confirmation that the protein BMP9 was absent in this individual.

His parents were each found to be heterozygous for the *GDF2* variant identified in their son. Neither parent had telangiectases or other cutaneous vascular lesions on examination, and both denied epistaxis. A targeted three generation family history was also negative for epistaxis or other history suggestive of HHT.

Individual 2: (NM_016204.3:c.[835G>T];[835G>T] or p.[Glu279Ter];[Glu279Ter]) was a 9‐year‐old female who presented with symptoms of a viral respiratory illness, and on admission was noted to be hypoxemic but otherwise did not appear to be acutely unwell. Her mother commented that her daughter's fingers had been “blue tinged” for several weeks. Her saturations on room air were 88%. Heart sounds, electrocardiogram, and chest X‐Ray were all normal and auscultation of her chest was unremarkable; in particular, no bruits could be heard. Hemoglobin level was elevated at 170 g/dL (normal 120–150 g/dL).

Multiple spider‐like and linear telangiectases were noted on the cheeks and chin, but not the lips or oral cavity. These telangiectases had first been documented at 3 years of age. On direct questioning she had a history of infrequent epistaxis occurring every few months. Her parents and brother were well although her brother also had a history of infrequent epistaxis. Of note, her parents were consanguineous.

**FIGURE 1:**
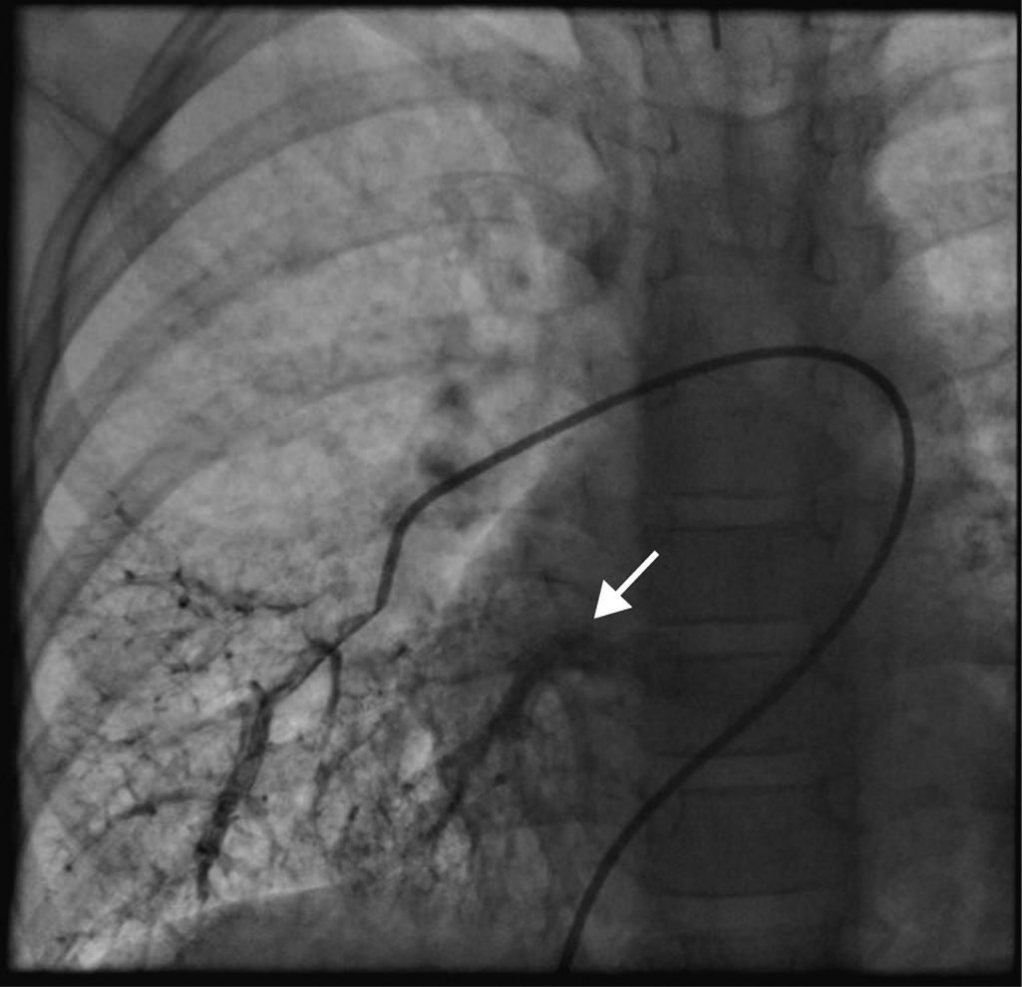
Pulmonary angiogram of right lower lobe in Individual 2. The angiogram indicates the location of multiple small pulmonary arteriovenous malformations with rapid venous return (arrow)

Given the cyanosis and telangiectases, a diagnosis of HHT with PAVM was considered and a CT pulmonary angiogram was performed. This showed no evidence of macroscopic PAVMs. The pulmonary artery and right‐sided heart chambers were unremarkable. Given the ongoing hypoxemia, a transthoracic contrast echocardiogram was then performed which demonstrated left atrial opacification after >3 cardiac cycles consistent with an intrapulmonary shunt, while no intra‐cardiac shunt could be visualized. Formal right heart catheterization and pulmonary angiography were therefore performed, revealing multiple small PAVMs in both lower lobes: the right middle lobe and the right upper lobe (Figure 1). There was rapid transit of contrast from the wedge pulmonary angiograms in these lobes into the pulmonary veins in around one cardiac cycle (arrow in Figure 1). The PAVMs were too small to be considered for percutaneous closure. Mean pulmonary arterial pressure was not elevated (10 mmHg). Magnetic resonance imaging of the brain and ultrasound of the liver did not demonstrate any other site of AVMs.

The diagnosis of HHT was initially suspected, but the atypical nature of the telangiectases and the infrequent nature of epistaxis means that only one of the four Curaçao criteria (visceral AVMs) were met (Shovlin et al., 2000). DNA sequence analysis of *ACVRL1*, *ENG*, and *SMAD4* was normal; large deletion/duplication analysis of *ACVRL1* and *ENG* was also normal. The patient was, however, homozygous for a non‐sense variant in the *GDF2* gene (c.[835G>T];[835G>T] or p.[Glu279Ter];[Glu279Ter]). Both parents were heterozygous for this variant, which was not present in the brother. Neither parent had any symptoms or clinical signs suggestive of HHT, and PAVMs were excluded by a negative bubble transthoracic echocardiogram in one parent and a negative thoracic CT scan in the other.

### 3.2 Measurement of plasma BMP9 and BMP10 levels and serum BMP activity

**FIGURE 2:**
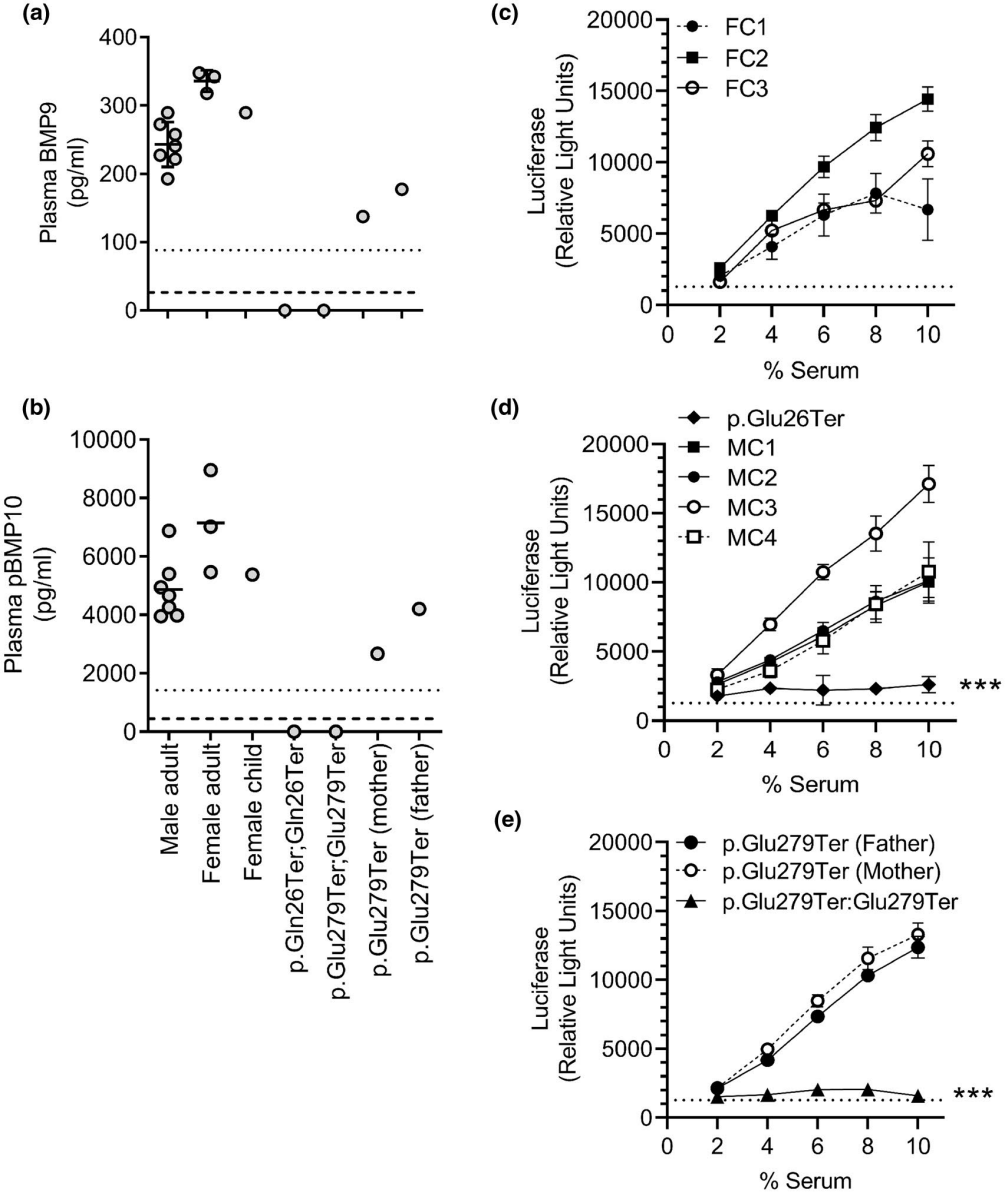
Homozygous *GDF2* truncation mutations lead to an absence of circulating BMP9 and pBMP10. (a,b) Levels of (a) BMP9 and (b) pBMP10 in plasma were measured with specific ELISAs. (a) Samples were assayed as 25% EDTA‐plasma, 0.2% goat‐serum, and 0.5% Triton X‐100 in BioRad ELISA buffer. (b) Samples were assayed as BMP9, but with inclusion of 4.5 mM EDTA. All samples were assayed in duplicate, each point represents a different individual. The dotted lines indicate the lower limits of quantification and dashed lines indicate the limits of detection for these assays. (c‐e) Endothelial serum BMP activity, representing BMP9 and BMP10, was assayed in the stable HMEC1‐BRE reporter line. Serum‐starved cells were incubated with 2%‐10% serum for 6 hr and luciferase activity then measured. Samples were measured in duplicate, and the assay was repeated on three separate occasions. Data are presented as mean of the averaged luciferase values for each individual experiment ± SEM. The dotted line indicates the mean SFM response. Individual graphs show: (c) control adult female samples from three separate individuals (FC1‐3), (d) p.[Gln26Ter]:[Gln26Ter] patient sample and control male samples from four separate individuals (MC1‐4), and (e) p.[Glu279Ter]:[Glu279Ter] patient sample and samples from unaffected p.[Glu279Ter] heterozygous parents. Data from all individuals were compared by a multiple comparisons two‐way ANOVA using GraphPad Prism 9. *p* < 0.001 is relative to all individual controls

As the termination mutations are predicted to cause an absence of the BMP9 growth factor domain (GFD; amino acids 320–429), we postulated that both homozygous truncating mutations would lead to a complete absence of circulating BMP9. Plasma BMP9 levels were measured in the p.[Gln26Ter];[Gln26Ter] and p.[Glu279Ter];[Glu279Ter] homozygotes, and the p.[Glu279Ter] heterozygous parents by ELISA (Figure 2a). As expected, there was no detectable circulating BMP9 in the homozygotes, while the heterozygotes had circulating levels below those of healthy controls, suggesting haplosufficiency. To maximize the sensitivity of this ELISA, we measured plasmas with a previously validated protocol (Hodgson et al., 2019) and also assessed increasing the plasma volume to 50 µL/well and mixing with a commercially available ELISA additive to reduce plasma matrix effects. In all cases, no BMP9 was detectable in the homozygous patients. We have recently reported a strong correlation between plasma BMP9 and pBMP10 (Pro:BMP10 and *ProBMP10*) levels (Hodgson et al., 2019). Therefore, we also measured pBMP10 levels in these patients and confirmed that individuals with homozygous *GDF2* mutations had low circulating pBMP10 levels (Figure 2b).

BMP9 and BMP10 are the main mediators of plasma/serum‐dependent activation of the Smad‐responsive BRE‐luciferase reporter in the HMEC1‐BRE reporter line (Hodgson et al., 2019). Sera from control females (Figure 2c) and males (Figure 2d) exhibited a linear increase in activity when incubated with HMEC1‐BRE cells over a range of 2%–10% by volume. By contrast, a very low BRE‐luciferase response was observed when HMEC1‐BRE cells were incubated with sera from either homozygous patient, suggesting dramatically reduced activity (Figure 2d,e). Of note, although reduced BMP9 and BMP10 levels were measured in the heterozygous p.[Glu279Ter] parents, their serum BRE‐luc responses were similar to the control individuals.

### 3.3 Assessment of alternate codon utilization in the p.[Gln26Ter] BMP9 mutant

**FIGURE 3:**
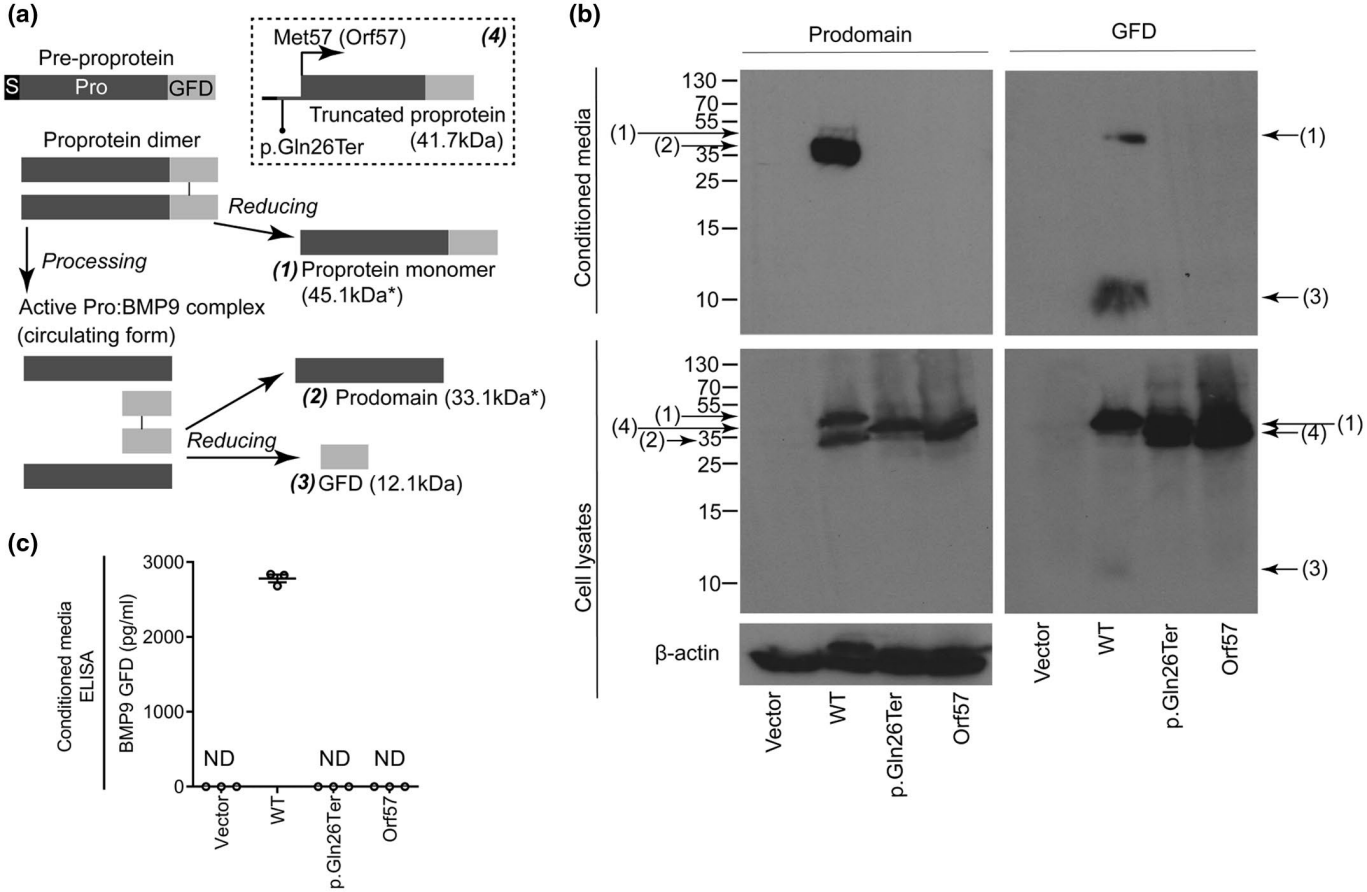
The BMP9 variant p.[Gln26Ter] can be expressed within cells, but is not processed or secreted. (a) Summary of the possible forms of BMP9 that may be produced. *ProBMP9* is cleaved into active Pro:BMP9 by furin‐like proteases during secretion (S, signal peptide; Pro, prodomain; GFD, growth factor domain). The dashed box contains the prediction of the p.[Gln26Ter] premature truncation mutation and predicted alternate translational initiation at Met57. The expected molecular weights of the proteins are noted in parentheses, though those secreted proteins that are glycosylated and may migrate at a larger molecular weight are denoted by (*). (b) HEK293 T cells were transfected with an empty vector, or expression vectors for *PreProBMP9*‐WT, p.[Gln26Ter] and BMP9 with the first 57 codons removed (Orf57). Cell lysates and conditioned media were fractionated by reducing SDS–PAGE, and immunoblotted for the BMP9 prodomain or growth‐factor domain. Equal loading of lysates was confirmed by subsequent immunoblotting for β‐actin. Numbers in parentheses refer to species shown in panel A. Blots are representative of n=3 separate experiments. (c) ELISA measurement of secreted BMP9 in conditioned media, the lowest standard detectable in conditioned media was 19.53 pg/ml (ND = not detected). Data in (b) are representative and (c) show individual values for proteins expressed on three separate occasions

BMP9 is initially produced as *PreProBMP9* from which the secretion signal sequence is cleaved to produce the immature *ProBMP9* dimer (Figure 3a). The polypeptide chain is then cleaved between the prodomain and GFD by furin‐type proteases to produce the active Pro:BMP9 complex (Bidart et al., 2012; Figure 3a). The p.[Gln26Ter] mutation occurs very early in the *GDF2* coding sequence, so we hypothesized that expression of the GFD might be rescued if ribosomes could initiate translation at Met57. To test this, we expressed plasmids encoding *PreProBMP9*‐WT, p.[Gln26Ter] and a version with the first 56 codons removed (designated Orf57). HEK293 T cell lysates were positive for both the prodomain and GFD from all these vectors, demonstrating that translation can be initiated at Met57 and a truncated protein results (Figure 3b). In contrast to the *ProBMP9*‐WT, which was processed to Pro:BMP9 and secreted into the media, the *ProBMP9*‐p.[Gln26Ter] and Orf57 were not processed or secreted, as no immunoreactivity was detected in conditioned media with an anti‐prodomain antibody (Figure 3b) or by ELISA (Figure 3c). These data are consistent with the loss of circulating BMP9 in p.[Gln26Ter] carriers and confirms that translational initiation at Orf57 does not lead to BMP9 secretion.

## 4 DISCUSSION

Previous reports of individuals homozygous for *GDF2* (encoding BMP9) variants have not confirmed that these mutations cause a loss of circulating BMP9. Here, we confirm that plasma BMP9 and pBMP10 levels and serum‐derived endothelial BMP activity are reduced in two individuals harboring homozygous *GDF2* mutations. One individual has PAH and the other has PAVMs, with cutaneous telangiectases common to both. Collectively, the cases presented here and previously published reports (Table 1) suggest that ultra‐rare homozygous *GDF2* mutations associate with a variety of pediatric presentations. Furthermore, a report of one affected child and an asymptomatic homozygous sibling in the same family suggests reduced and/or age‐related penetrance (Liu et al., 2020).

**TABLE 1:** Summary of reported *GDF2* homozygous pathogenic variant cases and clinical findings.

Protein change	Nucleotide change	Clinical findings (age at diagnosis)	Family history	Reference
p.[Gln26Ter]; [Gln26Ter]	c.[76C>T]; [76C>T]	‐PAH (3 years) ‐facial spider‐like/linear telangiectases (10 years)	Parents: both heterozygous, asymptomatic	Wang et al., (2016), this study
p.[Arg151Ter]; [Arg151Ter] *	c.[451C>T];[451C>T]	‐NIHF ‐Lymphatic dysplasia (34 + 6 weeks gestation)	Parents: both heterozygous, no suspicious findings 1. Sibling, intra‐uterine death due to NIHF 2. Unaffected heterozygous siblings	Aukema et al., (2020)
p.[Glu279Ter]; [Glu279Ter]	c.[835G>T];[835G>T]	‐facial spider‐like/linear telangiectases (3 years) ‐PAVMs – diffuse, small (9 years	Parents: both heterozygous, no suspicious findings	This study
p.[Tyr354ArgfsTer15]; [Tyr354ArgfsTer15]^a^	c.[1060_1062delinsAG]; [1060_1062delinsAG]	‐Hypoxia (5 years) ‐PAVMs – diffuse, small (8 years) (Epistaxis – reported as every 2–5 months at age 8 years, had ceased by age 9 years)	Parents: both heterozygous, no suspicious findings 1. Homozygous sibling: 7 years, unaffected (Vascular lesion on forehead)	Liu et al., (2020)

Note

This table summarizes the phenotypes of individuals homozygous for *GDF2* pathogenic variants currently known. GenBank reference sequence = NM_016204.3.

Abbreviations: HHT, hereditary hemorrhagic telangiectasia; NIHF, nonimmune hydrops fetalis; PAH, pulmonary arterial hypertension; PAVM, pulmonary arteriovenous malformations.

^a^ BMP9 protein levels have not been measured.

It should be emphasized that although the patients/families reported in this current study were initially suspected to have HHT, their cutaneous telangiectases are atypical in appearance and location for HHT. Both patients reported here had spider‐like/linear telangiectases only on their chin and/or cheeks. In stark contrast, telangiectases seen in HHT patients with *ACVRL1*, *ENG*, or *SMAD4* mutations are overwhelmingly punctate (not spidery) and typically occur on the lips and oral cavity, not other aspects of the face. A lack of cutaneous or nasal telangiectases was also noted in the report by Liu et al. of a child with a homozygous *GDF2* mutation (p.[Tyr354ArgfsTer15]; [Tyr354ArgfsTer15]), who presented with hypoxia and PAVMs (Liu et al., 2020). Of note, the PAVMs reported to date in patients with homozygous *GDF2* mutations have been small (tiny even) and diffuse, such that they were detectable by angiography, but not CT imaging (Liu et al., 2020). This is not common in HHT patients, although not unreported. Even the pediatric presentation in these family probands is not common in HHT. As children are most often minimally, if at all, symptomatic with HHT, the initial diagnosis is typically in an adult family member. Finally, none of the patients reported to date with homozygous *GDF2* mutations, nor their relatives, have a noteworthy history of epistaxis – the most common and usually earliest symptom of HHT. The cases reported here and by Liu et al., (2020) combined with previous cases of individuals with heterozygous *GDF2* mutations (Wooderchak‐Donahue et al., 2013) suggest that *GDF2* mutations can cause a syndrome that resembles HHT, but is nonetheless distinct from HHT caused by mutations in *ACVRL1*, *ENG*, or *SMAD4*.

Heterozygous germline mutations in *GDF2* and *BMP10* have been identified in PAH patients (Eyries et al., 2019; Gräf et al., 2018; Hodgson et al., 2019; X. Y. Wang et al., 2019). These result in reduced plasma BMP9 (Hodgson et al., 2019; X. Y. Wang et al., 2019) and also substantially reduced plasma pBMP10 levels (Hodgson et al., 2019). Furthermore, the capacity to induce endothelial BMP9/10 signaling was reduced in plasma samples from PAH patients with heterozygous *GDF2* mutations (Hodgson et al., 2019). In this study, plasma BMP9 and pBMP10 levels were slightly reduced in the asymptomatic p.[Glu279Ter] heterozygote parents, yet their serum activities were comparable to control sera. Therefore, reduced endothelial BMP activity associated with PAH in those heterozygous for *GDF2* mutations may represent genetic reduction of BMP9 combined with somatic reduction of BMP10 (Hodgson et al., 2019).

We observed a dramatic reduction of plasma BMP9 and pBMP10 levels and serum endothelial activity in both *GDF2* homozygous patients. The mechanism of the pBMP10 reduction in these individuals is not clear. It may support recent studies suggesting that BMP9:BMP10 heterodimers contribute to circulating activity (Tillet et al., 2018) or alternatively, if BMP9 and BMP10 are secreted by the liver and right atrium respectively, that some co‐regulation exists. In PAH patients without *GDF2* mutations, a few individuals have low BMP9 levels but detectable pBMP10 and vice‐versa, so heterodimers may not account for all circulating BMP9 and BMP10 (Hodgson et al., 2019). Accordingly, plasma Bmp10 can still be measured in *Bmp9* ^−/−^ mice, and Bmp9 can be measured in conditional *Bmp10* knockouts (Tillet et al., 2018). It would be interesting to measure BMP9 and pBMP10 levels and serum activity in the affected and unaffected siblings reported by Liu et al., (2020) to establish if a disease‐related difference is evident.

Disruption of the BMP9/BMP10—ALK1/BMPR‐II signaling axis seems to underlie many cases of human PAH (Atkinson et al., 2002; Hodgson et al., 2019; Morrell et al., 2019) and is implicated in the pathogenesis of HHT caused by germ‐line *ACVRL1* and *ENG* mutations (Kritharis et al., 2018). The association of *GDF2* mutations and reduced circulating BMP10 with either PAH or PAVMs suggests that impaired BMP9 and BMP10 signaling specifically impacts on the pulmonary vasculature. This is consistent with reports of reduced circulating BMP9 and BMP10 levels in portopulmonary hypertension patients (Nikolic et al., 2019; Owen et al., 2020; Rochon et al., 2020). Intriguingly, a recent report has suggested that BMP9 and BMP10 are not the “hepatic factor” preventing the development of PAVMs (Capasso et al., 2020). The PAVMs we observe in the homozygous p.[Glu279Ter];[Glu279Ter] patient combined with recent reports of PAVMs in a p.[Tyr354ArgfsTer15];[Tyr354ArgfsTer15] homozygote (Liu et al., 2020) and a p.[Gly291Ser] heterozygous mutation carrier (Topiwala et al., 2020) suggest that loss of BMP9 and BMP10 predispose individuals to the development of PAVMs. To date, studies of compound *Bmp9*:*Bmp10* knockout mice have not specifically noted AVM development, although dilated vessels associated with altered vascular smooth muscle cell functionality have been reported (L. Wang et al., 2021). It remains to be determined whether additional events are required to promote PAVM formation, either via disruption of BMP signaling, or by affecting other signaling pathways.

Our observation that homozygous germline *GDF2* mutations may cause either PAH or PAVMs similar to those found in HHT is not implausible. Heterozygous *ACVRL1* mutations generally cause the classic telangiectases and AVMs of HHT, but in rare cases can also cause PAH. The relative prevalence of HHT is 125–200 cases/million (Shovlin, 2014), with *ACVRL1* mutations accounting for 44% of affected individuals (55–88 cases/million; Kritharis et al., 2018). The prevalence of Group I PAH is 15–26 cases/million (Archer et al., 2010) and *ACVRL1* mutations constitute approximately 1% of cases (Machado et al., 2015), so *ACVRL1* mutations are most frequently causal of HHT. However, on rare occasions, individuals with *ACVRL1* mutations exhibit PAH without HHT (Chida et al., 2012; Fujiwara et al., 2008) or a combined syndrome of PAH and HHT (Chida et al., 2012; Trembath, 2001). Furthermore, Harrison et al. reported that a PAH patient harboring a missense *ACVRL1* mutation had no family or personal history of HHT, nor any evidence of HHT at necropsy (Harrison et al., 2003). Also, in 7 out of 9 individuals with *ACVRL1* mutations in the French registry of PAH patients, the diagnosis of PAH preceded that of HHT (Girerd et al., 2010), suggesting that *ACVRL1* mutations may predispose to HHT or PAH. Similarly, *GDF2* mutations may predispose to PAH (Eyries et al., 2019; Gräf et al., 2018; Hodgson et al., 2019; G. Wang et al., 2016; Wang et al., 2019) as well as an “HHT‐like” syndrome characterized by cutaneous telangiectases and AVMs (Liu et al., 2020; Wooderchak‐Donahue et al., 2013). Intriguingly, a recent report of a family with a child with non‐immune hydrops fetalis, hydrothorax, and lymphatic dysplasia has identified a homozygous *GDF2* (p.[Arg151Ter];[Arg151Ter}) mutation (Table 1; Aukema et al., 2020). Again, protein levels were not determined, so it is not known if this mutation is subject to, or escapes from nonsense‐mediated decay (Lappalainen et al., 2013). Nonsense‐mediated decay is inefficient when premature truncation codons are located downstream of the last exon junction complex (Lindeboom et al., 2016), so one might predict secretion of a truncated prodomain and no growth factor. If all of the identified homozygous nonsense mutations cause BMP9 loss, one might then speculate that the bias toward a particular syndrome is driven by modifier genes, epigenetic changes, somatic events, or a combination of these.

In conclusion, these observations suggest that a diagnostic gene panel tests for those with pediatric PAH, PAVMs, and/or cutaneous telangiectases should include *GDF2* and by association, *BMP10*. This because the phenotypes caused by mutations in *GDF2* and *ACVRL1*, *ENG*, or *SMAD4* are clearly overlapping. That said, as compared to accepted features of HHT associated with mutations in these other three genes, individuals described to date with mutations in *GDF2* have a distinctly different appearance and pattern to their cutaneous telangiectases, a presentation of PAVMs noteworthy for being tiny but diffuse, and are relatively more likely to have PAH.

## CONFLICT OF INTERESTS

PDU is a founder and scientific advisor to Morphogen‐IX Ltd. NWM is a founder and CEO of Morphogen‐IX Ltd. PDU and have published US (US10336800) and EU (EP3166628B1) patents entitled: “Therapeutic Use of Bone Morphogenetic Proteins.” All other authors declare that they have no competing interests.

## AUTHOR CONTRIBUTIONS

Jamie McDonald, Oliver Quarrell, Pinar Bayrak‐Toydemir, Whitney Wooderchak‐Donahue, Robin Condliffe, Carmelo Bernabeu, Paul D Upton carried out conceptualization; Joshua Hodgson, Lidia Ruiz‐Llorente, Oliver Quarrell, Kelechi Ugonna, James Bentham, Rebecca Mason, Jennifer Martin, David Moore, Katie Bergstrom, Pinar Bayrak‐Toydemir, Whitney Wooderchak‐Donahue, Robin Condliffe, Carmelo Bernabeu, Paul D Upton performed data curation; Joshua Hodgson, Lidia Ruiz‐Llorente, Jamie McDonald, Robin Condliffe, Carmelo Bernabeu, Paul D Upton performed formal analysis; Joshua Hodgson, Lidia Ruiz‐Llorente, Robin Condliffe, Carmelo Bernabeu, Paul D Upton performed methodology; Joshua Hodgson, Lidia Ruiz‐Llorente, Jamie McDonald, Oliver Quarrell, David Moore, Katie Bergstrom, Robin Condliffe, Carmelo Bernabeu, Paul D Upton performed validation; Joshua Hodgson, Lidia Ruiz‐Llorente, Jamie McDonald, Oliver Quarrell, Kelechi Ugonna, James Bentham, David Moore, Katie Bergstrom, Pinar Bayrak‐Toydemir, Whitney Wooderchak‐Donahue, Nicholas W Morrell, Robin Condliffe, Carmelo Bernabeu, Paul D Upton carried out investigation; Joshua Hodgson, Lidia Ruiz‐Llorente, Jamie McDonald, Robin Condliffe, Carmelo Bernabeu, Paul D Upton were involved in writing—original draft preparation; Jamie McDonald, Oliver Quarrell, Pinar Bayrak‐Toydemir, Whitney Wooderchak‐Donahue, Nicholas W Morrell, Robin Condliffe, Carmelo Bernabeu, Paul D Upton; Visualization, Jamie McDonald, Nicholas W Morrell, Robin Condliffe, Carmelo Bernabeu, Paul D Upton; Supervision, Nicholas W Morrell, Robin Condliffe, Carmelo Bernabeu, Paul D Upton did writing—review and editing; Rebecca Mason and Jennifer Martin project administration,; Funding acquisition, Nicholas W Morrell, Carmelo Bernabeu, Paul D Upton. All authors have read and agreed to the published version of the manuscript.

## Data Availability

The new genetic data that support the findings of this study are openly available in LOVD3 at databases.lovd.nl/shared/variants, reference number 0000703987. All other data that support the findings of this study are available from the corresponding author upon reasonable request.
